# Genome-wide association study of agronomic traits in rice cultivated in temperate regions

**DOI:** 10.1186/s12864-018-5086-y

**Published:** 2018-09-25

**Authors:** Juan L Reig-Valiente, Luis Marqués, Manuel Talón, Concha Domingo

**Affiliations:** 1 Centro de Genómica, Instituto Valenciano de Investigaciones Agrarias, Carretera CV 315 Km 10,7, 46113 Moncada, Spain; 2 Cooperativa de Productores de Semillas de Arroz, Sueca, Spain

**Keywords:** Rice, GWAS, Yield, Heading date

## Abstract

**Background:**

Rice plants are sensitive to the agro-climate conditions, being photoperiod one of main factor contributing to their adaptation to the region where they are grown. Dissecting the genetic bases underlying diversity in rice populations adapted to specific environmental conditions is a fundamental resource for breeding. In this study we have analysed a collection of *japonica* varieties adapted to temperate regions to perform association studies with traits of high agronomical interest such as heading date, plant height, number of panicles, panicle length and number of grains per panicle.

**Results:**

We have performed a genome wide association study using a panel of 1713 SNPs that, based on previous linkage disequilibrium estimations, provides a full coverage of the whole genome. We have found a total of 43 SNPs associated with variations in the different traits. The identified SNPs were distributed across the genome except in chromosome 12, where no associated SNPs were found. The inspection of the vicinity of these markers also revealed a set of genes associated with physiological functions strongly linked to agronomic traits. Of special relevance are two genes involved in gibberellin homeostasis that are associated with plant height and panicle length. We also detected novel associated sites with heading date, panicle length and number of grain per panicle.

**Conclusion:**

We have identified loci associated with important agronomic traits among cultivars adapted to temperate conditions. Some of these markers co-localized with already known genes or QTLs, but the association also provided novel molecular markers that can be of help to elucidate the complicated genetic mechanism controlling important agronomic traits, as flowering regulation in the non-dependent photoperiod pathway. The detected associated markers may provide important tools for the genetic improvement of rice cultivars in temperate regions.

**Electronic supplementary material:**

The online version of this article (10.1186/s12864-018-5086-y) contains supplementary material, which is available to authorized users.

## Background

One of the main challenges for rice breeders is to develop high yielding cultivars. As rice is a staple food in many countries, high yield is required to provide food to a large and rising population. It is also desirable to increase the incomes of farmers and the economic profitability of the crop.

Since domestication, rice cultivation expanded towards different agro-ecological environments by the generation of new cultivars through selection of adapted plants to the new conditions through intense and continuous breeding activities [8]. One of the barriers that rice overcame to reach temperate regions was the difference in day length that became one of the main determinants of plant adaptation to new areas [10, 11]. As a consequence, the genetic distance between cultivars from tropical regions and those cultivated in temperate regions enlarged enough to produce strong reproductive barriers that hinder the genetic flow between tropical and temperate cultivars [12] and contributing to the constriction of the genetic pool of cultivars and the emergence of different subpopulations. The two main varietal cultivated groups, *indica* and *japonica*, are characterized by adaptations to specific climates, according to the agro-ecological conditions where they were cultivated. *Indica* cultivars are grown in tropical latitudes, whereas *japonica* cultivars can be found either in tropical or temperate climates [21]. Due to the adaptation of cultivars to specific climates, in particular to the local photoperiod, the use of cultivars from different subpopulation as donors in breeding programs is challenging since it encompasses the introduction of undesirable traits that may not be appropriate for the specific climate requirements.

The area where *japonica* type is cultivated is wide enough to hold relevant natural diversity covering a wide spectrum of morphological and physiological variations [24]. The characterization of the genetic bases of this diversity will allow the identification of loci that underlie this phenotypic variation, especially those concerning agronomic traits, with direct application to breeding. This achievement will offer novel opportunities to select appropriated donors to incorporate new traits of interest into local cultivars while conserving those characters responsible for temperate climate adaptation.

Yield is a complex trait that involves multiple component traits and depends on several genetic and environmental factors [34]. Adaptation of cultivars to specific agro-ecological conditions is an essential condition for high yield and, in this sense, an optimal heading date is necessary. The number of grains that a plant may produce is also determinant and it can vary according to the number of grains per panicle and the number of panicles, which are quantitative traits [34]. Panicle number is a difficult trait to study as it relays not only on genetic factors but also on environmental and growth conditions as the density of plants in the fields. Other morphological factors as height and panicle length also affect final yield.

Taking advantage of the diversity of rice, based on historic recombination events and linkage disequilibrium across the genome, genome wide association studies (GWAS) have recently become popular to identify QTLs in plant populations. Several GWAS have been performed in a search for genes involved in traits affecting yield. Studies on panicle architecture, number of spikelets, grain size or heading date have been reported [1, 23]. Most of them have been conducted in *indica* populations or comparing *indica* and *japonica* rice or other wild rice species [9, 23, 38]. A few studies have also been performed to detect associations within *japonica* populations [32, 36]. Still, the genetic basis of the phenotypic variations among temperate cultivars needs further analysis.

In a previous work, we generated a collection of rice cultivars that represented the genetic diversity present in temperate regions possessing an enormous variety of agromorphological and physiological characters. The collection is composed by 193 traditional landraces and modern elite and old cultivars of rice. The study of the population structure and the genetic relationship among the cultivars evidenced a strong substructure in the temperate rice collection, predominantly based on grain type and the origin of the cultivars [24]. We observed the occurrence of relatively high gene flow and elevated rates of admixture between cultivars grown in remote regions, probably favoured by local breeding activities. The results enable genome-wide association studies of complex traits and functional gene investigations among cultivars acclimated to temperate regions preventing the generation of spurious associations due to subpopulation structure and unknown relationships between cultivars.

In this study, the genetic bases underlying complex agronomic traits contributing to high yield in *japonica* temperate rice were investigated with the purpose of facilitating rice breeding in temperate regions. We conducted a genome-wide association study and identified and located several QTLs for the investigated traits.

## Results

### Phenotyping evaluation

**Fig. 1:**
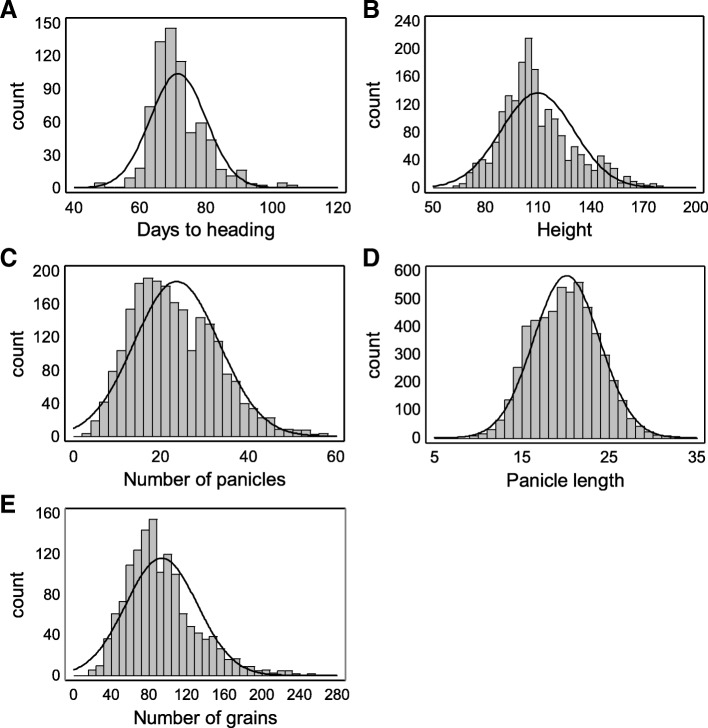
Phenotypic distributions for the studied agronomic traits. (**a**) heading date, (**b**) plant height, (**c**) number of panicles, (**d**) panicle length and (**e**) number of grains per panicle. Histograms show data of all plants used in the assays. The resulting curves from fitting the data to a normal distribution are shown

Plants from a collection of 193 *japonica* cultivars adapted to temperate regions were cultivated and evaluated during two consecutive years in natural long day field conditions during summer season. All plants were able to flower under these photoperiod conditions. Heading date (DH) of the varieties in the collection ranged from108 to 47 days (Table 1). Plant height (H), number of panicles (PN) and panicle length (PL) were scored (Additional file 1: Table S1). Traits presented large phenotypic variation (Table 1) with coefficient of variation ranging from 0.12, in the case of heading time, to 0.41 in the number of panicles. All the measured variables showed broad sense heritability values (Table 1). Height varied from 182 to 62 cm, displaying the highest heritability value, 0.92. Panicle length ranged from 34.4 to 9.6 cm showing also a high heritability, 0.81.PN varied from 59 to 4 panicles per plants showing the lowest heritability value, 0.54. Distributions of phenotype frequencies in general displayed an approximately normal distribution (Fig. 1), although only panicle length frequencies were normally distributed. Panicle number data collected in Malta field, during 2016 were excluded from the analysis due to differences in scoring between the individual locations and years, showing significantly lower values than the other assays (Additional file 2: Figure S1). As some of the cultivars displayed dehiscence, the number of grains per panicle (GN) was scored in two batches of plants grown in a greenhouse under natural day conditions before they reached full maturity when data was collected.

**Table 1:** Phenotypic variations of the studied traits

Trait	Mean	Minimum	Max	SD	CV	H2
Height (cm)	109.7	62.0	182.0	20.8	0.19	0.92
Days to heading	71.6	47.0	108.0	8.5	0.12	0.79
Panicle length (cm)	20.1	9.6	34.4	3.8	0.19	0.81
Grains per panicle	93.8	22.0	264.0	37.9	0.40	0.77
Number of panicles	23.6	4.0	59.0	9.7	0.41	0.54

Estimation of mean, minimum, maximum for days, standard deviation (SD) and coefficient of variation (CV) to heading, height, panicle length, number of grains per panicle, number of panicles and grain weight from all experiments. Broad sense heritability calculated for two years experiments

Correlations exhibited by the agronomic traits between each other are shown in Table 2. Panicle length exhibited high correlation with plant height. Days to heading showed moderate correlation with height and panicle length. Panicle length was no significantly correlated with number of grains. Other traits showed weak or no correlation among each other.

**Table 2:** Correlation coefficients of paired traits

Trait	height	days to heading	panicle length	grains per panicle
days to heading	0.42**			
panicle length	0.61**	0.41**		
grains per panicle	0.32**	0.29**	0.28**	
number of panicles	−0.30**	−0.02	− 0.21*	−0.24**

** significant at *P* < 0.001. * significant at *P* < 0.01

**Fig. 2:**
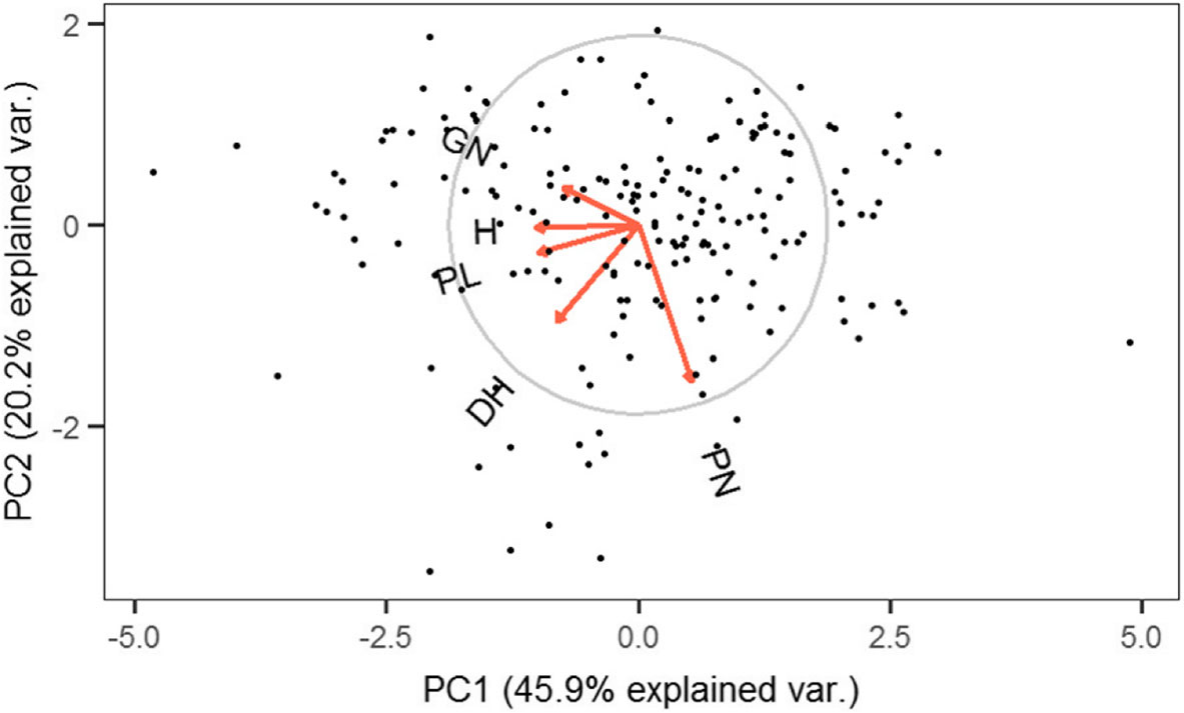
Principal components analysis (PCA) plots. The proportion of the variance explained by the first (PC1) and second principal (PC2) components is indicated in parenthesis. DH, heading date; H, plant height; PN, number of panicles; PL, panicle length and GN, number of grains per panicle

To identify the relation between traits, and to analyse the distribution of cultivars according to their agronomic characteristics, a principal component analysis (PCA) was performed using the phenotypic variables studied in this work. The first principal component (PC1), which accounted for 45.9% of the variance, separated cultivars according to panicle length, number of grains per panicle and plant height (Fig. 2). This is in accordance with the correlation observed between these traits shown in Table 2. On the other side, PC2, explaining 20.1% of the variance distinguished cultivars according to number of panicles.

### Association analysis

**Fig. 3:**
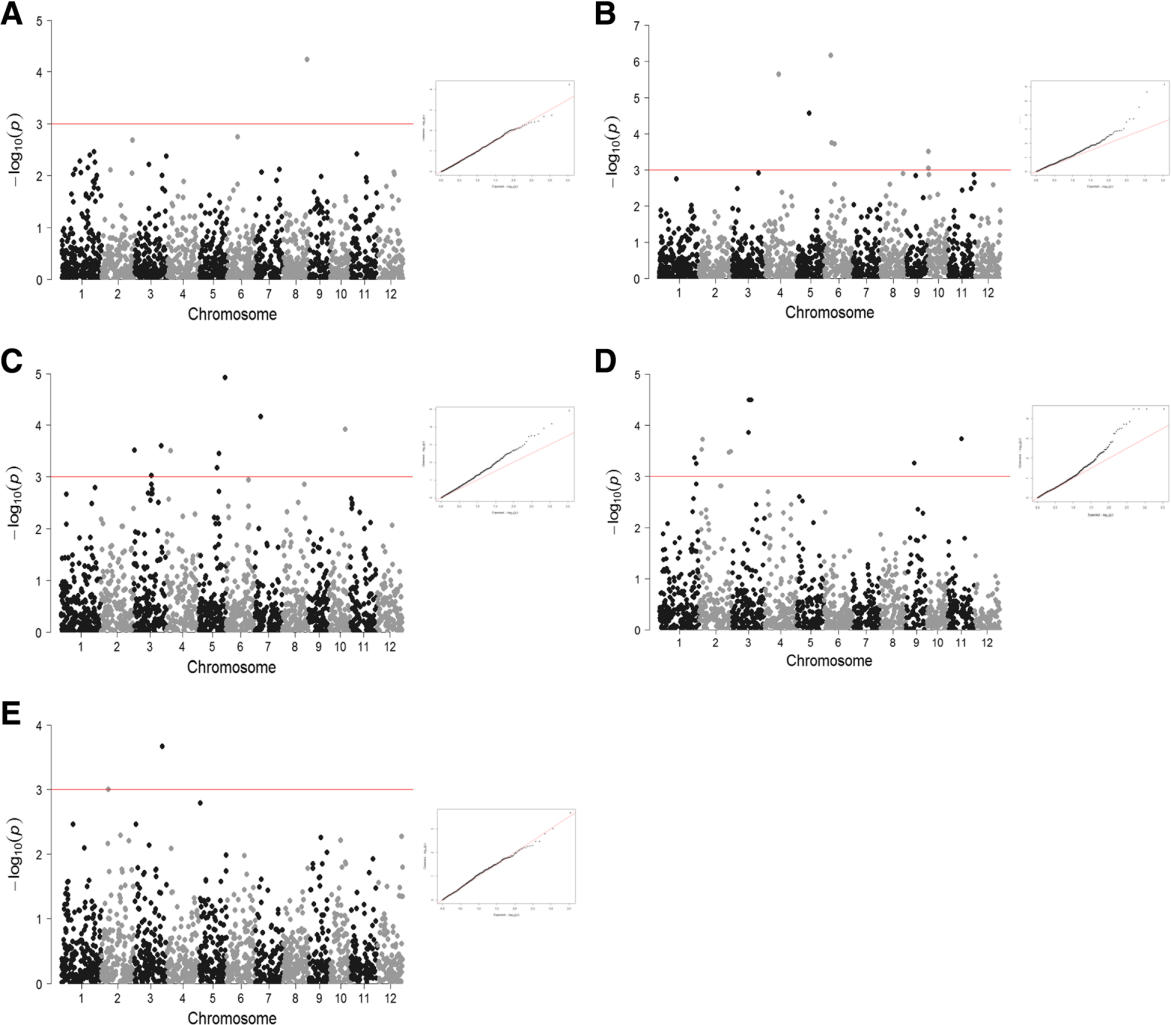
Genome-wide association mapping of the traits in this study. Manhattan and quantile-quantile plots for (**a**) plant height, (**b**) heading date, (**c**) panicle length, (**d**) number of grains per panicle and (**e**) number of panicles. The red horizontal line indicates genome- wide significant threshold

To detect associations between SNP markers and variations in the evaluated traits in the 193 *japonica* cultivars in the collection, we used the genotypic dataset generated in a previous study consisting in a panel of 1713 SNPs uniformly distributed across the whole genome with a mean distance of 215 Kb between each other [24]. According to the linkage disequilibrium estimated for this collection, 368 kb in average, [24] and the rice genome size, 321 Mb [13] the number of SNPs in the panel is adequate to detect associations across the whole genome. Among several available linear models, we chose the Mixed Linear Model (MLM) as it produces fewer spurious associations than other methods [41]. It has been suggested that in the case of rice, MLM reduces the number of false positive but in contrast increases the number of false negatives, overcompensating for population structure and relatedness [43]. We have previously shown that our temperate rice collection has a strong structure displaying several subpopulations [24]. Therefore, to avoid false associations due to the population genetic structure, we used two approaches, a first analysis considering the population structure given by the STRUCTURE software [24] and a second association study based on the population structure according to a PCA analysis (Fig. 2). We set a *p*-value < 0.001 threshold to consider a SNP to be significantly associated with the trait variation. In addition, q-values were also estimated [28]. The quantile-quantile (QQ) plots and Manhattan plots for the analysed traits obtained using the STRUCTURE Qmatrix are shown in Fig. 3. QQ plots for heading date, panicle length and number of grains indicated that the model was well fitted to the data; the observed *p*-values were uniformly distributed with some deviation at high values from the expected *p*-values.

**Fig. 4:**
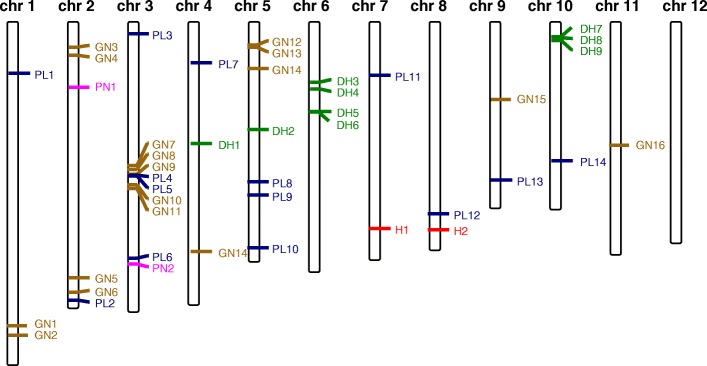
Physical map position of significant associated SNPs detected in the GWAS. The identified sites are labelled in dark green for heading date (DH), in red for height (H), in blue for panicle length (PL), in orange for number of grains per panicle (GN) and pink for number of panicles (PN)

The statistical analysis revealed 43 SNPs significantly associated with the different traits (Table 3). The identified SNPs were distributed across the genome except in chromosome 12, where no associated SNPs were found (Fig. 4). The explained variance, given by R^2^ ranged between 5.2 and 14.3%. Some identified SNPs were corroborated by the two approaches, according to the population structure defined by STRUCTURE software or to the PCA analysis, both approaches shared 18 SNPs with a p-value lower than the threshold set for significance. Considering an association locus as a chromosomal region in which the distance between the adjacent pairs of associated SNPs is given by the estimated LD, these SNPs could be grouped in 33 loci associated with the studied traits (Fig. 4, Table 3).

**Table 3:** List of SNPs significantly associated with the studied traits

				structure			PCA		
SNP	Loci number	Chr	Pos	*p*-value	*q*-values	% explained variance	*p*-value	*q*-values	% explained variance
H-1	1	7	26,125,810				8.53E-04	0.691	5.5
H-2	2	8	26,290,663	5.74E-05	0.098	8.0	1.54E-05	0.026	9.0
DH-1	3	4	15,171,082	2.33E-06	0.002	14.3	1.01E-05	0.009	12.5
DH-2	4	5	13,373,075	2.76E-05	0.014	8.9	6.75E-05	0.031	8.1
DH-3	5	6	7,249,825	6.91E-07	0.001	12.4	1.67E-07	0.000	13.5
DH-4	6	6	8,122,336	1.82E-04	0.058	7.2	7.18E-05	0.031	8.0
DH-5	7	6	11,002,220				7.12E-04	0.185	7.4
DH-6	7	6	11,018,229	1.94E-04	0.058	7.1	9.60E-05	0.033	7.7
DH-7	8	10	1,366,920	9.09E-04	0.181	7.2			
DH-8	8	10	1,500,932	3.10E-04	0.078	6.7			
DH-9	8	10	1,871,964				7.59E-04	0.185	5.8
PL-1	9	1	6,126,957				7.34E-05	0.054	7.7
PL-2	10	2	35,406,962				6.78E-04	0.119	5.8
PL-3	11	3	1,003,670	3.09E-04	0.081	6.7	6.48E-04	0.119	5.9
PL-4	12	3	19,250,700				6.99E-04	0.119	7.2
PL-5	12	3	19,349,943	9.37E-04	0.169	8.1	5.93E-04	0.119	8.5
PL-6	13	3	29,987,079	2.49E-04	0.081	6.8	8.55E-04	0.131	5.5
PL-7	14	4	4,758,113	3.15E-04	0.081	6.6	1.98E-04	0.084	6.9
PL-8	15	5	20,125,768	6.72E-04	0.136	5.9			
PL-9	15	5	21,838,857	3.52E-04	0.081	6.5			
PL-10	16	5	28,626,704	1.21E-05	0.020	11.3	1.43E-05	0.024	10.8
PL-11	17	7	6,375,823	6.80E-05	0.055	9.7			
PL-12	18	8	24,250,565				3.15E-04	0.108	8.0
PL-13	19	9	19,877,778				9.50E-05	0.054	7.5
PL-14	20	10	17,376,318	1.19E-04	0.064	9.2	4.78E-04	0.119	7.6
GN-1	21	1	38,011,958	4,33E-04	0,064	6,4			
GN-2	21	1	40,012,174	5,64E-04	0,070	6,1			
GN-3	22	2	2,720,687	2,99E-04	0,056	6,7			
GN-4	22	2	3,755,851	1,89E-04	0,044	7,1			
GN-5	23	2	32,500,801	3,46E-04	0,056	8,1			
GN-6	23	2	34,386,360	3,25E-04	0,056	6,6			
GN-7	24	3	17,999,538	1,39E-04	0,044	7,4			
GN-8	25	3	18,500,293	3,23E-05	0,013	8,7	9,63E-04	0,197	5,2
GN-9	26	3	19,125,149	3,23E-05	0,013	8,7	9,63E-04	0,197	5,2
GN-10	27	3	20,500,343	3,23E-05	0,013	8,7	9,63E-04	0,197	5,2
GN-11	28	3	20,875,010	3,23E-05	0,013	8,7	9,63E-04	0,197	5,2
GN-12	28	5	2,460,569				7,67E-04	0,197	5,2
GN-13	28	5	2,753,203				1,48E-04	0,197	6,6
GN-14	29	5	6,499,710				9,98E-04	0,197	6,5
GN-15	30	9	9,375,311	5,58E-04	0,070	6,2	9,06E-04	0,197	5,4
GN-16	31	11	15,123,344	1,87E-04	0,044	7,2			
PN-1	32	2	7,879,224	9.94E-04	0.710	5.5			
PN-2	33	3	30,752,398	2.15E-04	0.318	6.9	1.20E-04	0.171	6.5

Associated SNPs to height (H), days to heading (DH), Panicle length (PL), number of grains per panicle (GN) and number of panicles (PN) according to population structure given by STRUCTURE program or by PCA analysis

Among the associated sites, nine SNPs were significantly associated to heading date (Table 3) of which seven were novel sites. Some associated markers displayed promising allele distribution among the cultivars (Additional file 3: Table S3). This is the case of DH-8. Accessions carrying different alleles of SNP DH-2, localised at position 13,373,075 in chromosome 5, flowered on average 17 days earlier than accessions carrying the alternative allele (Additional file 4: Table S2). The mean values for heading date of accessions carrying different alleles at DH-7, at position 1,366,920 in chromosome 10 was 7 days at flowering (Additional file 4: Table S2). Additionally, for the 20 earliest cultivars, 19 were confirmed to carry the G allele at DH-7 position, meanwhile for the 20 latest ones 14 carried the A allele. We looked for functional genes related to the traits in the vicinity of the SNP in an extended the interval of 368 Kb according to the estimated LD decay. DH-3 and DH-4 co-localized with previously described genes or QTLs. Two QTLs, *photoperiod sensitive phase 6* (*qPSP-6)* and *qDTH6*, involved in the regulation of flowering mediated by the photoperiod [3, 5] showed positional relationship with DH-3, located at position 7,249,825. Additionally, DH-3 is relatively close, less than 1 Mb, to *Su-Se1* (Table 4), a QTL that has also been implicated in the photoperiodic regulation of flowering [40]. The mean value for heading date of accessions carrying different DH-3 alleles showed a difference of 5 days at flowering (Additional file 4: Table S2). For the 20 earliest cultivars, 13 were confirmed to carry the G allele at DH-3 position meanwhile 19 cultivars corresponding to the 20 latest cultivars also carried the same allele.

**Table 4:** List of known genes or QTLs related to the traits within 0.36 Mb distance interval

SNP	QTL / gene	QTL/gene name	locus	chr	position	reference
H-1	*DEP2*	*cleistogamy gene*	Os07g0616000	7	26,041,969–26,049,689	[16]
DH-3	qPSP-6	photoperiod sensitive phase 6	–	6	6,720,901–8,066,362	Cai et al. 1998
DH-4	qDTH-6	–	–	6	8,054,255–8,066,362	Fujino et al. 2005
DH-4	Su-Se-1(t)		–	6	8,054,255–8,066,362	[40]
PL-6	*rip-3*	*rice panicle 3* (α-tubulin)	Os03g0726100	3	30,288,045–30,290,829	[27]
PL-8	*sdg / gid1*	*semidwarf gene g / gibberellin insensitive dwarf1*	Os05g0407500	5	19,875,232–19,878,096	[31]; Zhang et al. 2012
PL-13	*LGD1*	*Lagging Growth and Development 1*	Os09g0502100	9	20,078,670–20,084,674	[30]
PL-14	qssd10	spikelet setting density 10	–	10	12,044,545–19,623,828	[33]
GN-3, GN-4	*LRK1*	*leucine-rich repeat receptor-like kinase1*	Os02g0154200	2	2,978,853–2,982,354	Za et al. 2009

Panicle length showed the highest number of associated markers, 14 markers that were distributed across 9 chromosomes (Fig. 4). Five of these markers co-localized with genes involved in plant height or panicle length. PL-6, in chromosome 3, co-localised with *rice panicle 3* (*rip-3*), coding for an α-tubulin that probably participates in the suppression of the panicle elongation during water deficit [27]. *Semidwarf gene g / gibberellin insensitive dwarf1* (*sdg / gid1*) co-localised to PL-8, in chromosome 5 (Table 4). Mutants carrying a null allele of *gid1* showed dwarf phenotype [29, 31]. Allele A at PL-8 position was present in 17 cultivars of the corresponding 20 with the shortest panicles. On the other hand, allele G was present in 17 of the 20 cultivars with the longest panicles (Additional file 4: Table S2). In a similar manner, allele G at PL-9, in the same locus that PL-8, was found in 17 of the 20 cultivars with the shortest panicles; meanwhile allele A was present in all cultivars corresponding to the 20 with the longest panicles. PL-13, associated with panicle in chromosome 9, co-localised with *LAGGING GROWTH AND DEVELOPMENT 1* (*LGD1*) implicated in growth and in the formation of panicle. The *lgd1* mutant showed altered panicle architecture [30]. Finally, QTL *ssd10* (*spikelet setting density*) is located at the same position as PL-14 in chromosome 10 [33].

We detected 16 significant markers associated with the number of grains per panicle. GN-3, in chromosome 2, co-localized with *leucine-rich repeat receptor-like kinase 1* (*LRK-1*) (Table 4). LRK1 is a plasma membrane protein that, supposedly, regulates rice branch number by enhancing cellular proliferation [42]. One marker, in chromosome 7 was identified associated to height. H-1 co-localized with *dense and erect panicle 2* (*DEP2*) [16]. *DEP2* is involved in the elongation of rachis and primary and secondary branches in the panicles and plants carrying mutations in DEP2 exhibited smaller panicles but also reduced height [16]. Finally, two additional markers, in chromosome 2 and 3, were associated with the number of panicles (Table 3). Neither QTLs nor candidate genes were found nearby these markers.

## Discussion

After domestication, despite the non-favourable flowering conditions, rice cultivation area expanded across regions with temperate climate. Modulating the sensitivity photoperiod, plants were able to overcome the inhibition of flowering during long day conditions and reached Northern latitudes. High summer temperatures, accompanied by higher number of hours of light with good solar radiation, constituted an excellent growth environment allowing expansion and originating wide diversity that is reflected in different agronomic traits, as it is shown in this study. The diversity in plants already adapted to the photoperiod is a useful resource for breeders as it may provide donors suitable for their local agronomical conditions. The majority of the analyses that have been previously reported on the identification of variations in genes of agronomic interest have been performed comparing *japonica* and *indica* cultivars. Thus, factors involved in phenotypic variations among *japonica* subpopulation need further investigation.

In this study, we have used GWAS as an effective method to detect associations between genomic regions and phenotypes [9, 19] of cultivars that are grown across regions with temperate climate. We have used a collection of cultivars, generated previously, including cultivars from 23 countries, that represents the genetic diversity present in temperate climate regions and constitutes a useful resource for the identification of genetic factors governing variations in agronomic traits in these regions. In fact, this collection has already been successfully proved to detect polymorphism associated with cold tolerance [25]. The analysis performed in this study has taken into account the structure of the population and relatedness among cultivars, two factors that may led to false associations in GWAS. We have considered two possibilities, one that distributed the collection into four sub-populations according to STRUCTURE software [24] and a second structure generated by PCA analysis. The analyses performed considering both Q matrix gave similar results, and most of the detected SNPs were identified using any of the both possibilities in the case of height, panicle length and panicle number (Table 3). It is remarkable that for DH-7, DH-8 and DH-9, which are positioned within an interval of 505 Kb, the association was detected using either one or the other structure considerations.

Flowering is a trait of great interest for breeders. An optimum heading date, appropriated to the day length in a local region, is necessary for a maximum yield. Control of flowering by photoperiod has supposedly been one of the main factors that contributed to the expansion of rice through regions with long day conditions. It is also a key factor in the adaptation of cultivars to each local condition and it is determined by the latitude where they are grown. Regulation of flowering by the photoperiod has been extensively studied and, nowadays, it is already known that the fine-tuning regulation of *Hd3a* and *RFT1* promotes flowering under short or long day conditions [15]. While *Hd3a* dictates the transition of rice from vegetative to reproductive stage under short day conditions*, RFT1* has been pointed as the major floral activator under long day conditions. Winter cold temperatures in temperate climate regions constrain rice cultivation, preventing two harvests per year even in the case of early flowering. But shortening the growth duration in a few days is still desirable by farmers as it increases crop security by reducing the risk of pathogen attacks or weather adverse conditions like storms at the end of the growing season, frequent in the East coast of Spain. All cultivars in our temperate collection were able to flower under natural long day conditions and heading date varied between 47 and 106 days (Table 1). Nine loci were found to be associated with heading date. In this study we expected to find the source of heading date variation among cultivars already adapted to long days, originated in areas with little photoperiod differences. But, surprisingly, one marker in chromosome 6, DH-4, co-localised with *Su-Se-1* and qDTH6, QTLs that were identified in photoperiod regulation studies (Table 4). *Su-Se-1* is a dominant photoperiod-sensitive suppression gene identified in a cross between Asominori x IR24 [40]. qDTH6 was isolated in a cross between Hayamasari, an early cultivar from Japan, and Italica Livorno [5]. These authors suggested the possibility that *qDTH6* may be *Hd1*, a key regulatory gene in the photoperiodic control of flowering [37]. *Hd1* is located in chromosome 6, at a distance of 1.2 Mb from DH-4, a high distance for considering the association between both genomic regions. It has been reported that the allelic heterogeneity and complex genome structure of the *Hd1* causes spurious associations and a shift in the genomic position of the signal corresponding to *Hd1* has been observed in some association studies [36]. We have previously investigated the *Hd1* structure in 52 cultivars included in the present collection and found 12 different variants that include non-functional alleles of *Hd1* [20]. *HD1* is a repressor of flowering under long day conditions through the inhibition of *Hd3a* expression [14] and it has been suggested that the lack of functionality of *HD1* is crucial for the adaptation of rice to long day condition in temperate regions [7]. This hypothesis is reinforced by the fact that loss-of function alleles of *HD1* are common in cultivated rice in Northern latitudes ([6]; Gómez-Ariza et al., 2015). However, previous studies have concluded that HD1 is not involved in the regulation of flowering under long day conditions, as many cultivars grown in these conditions carried both functional and non-functional allele of HD1 independently of the geographical origin of the cultivars [20]. The possibility of the occurrence of another factor involved in flowering in the immediacy to *Hd1* should not be discarded and it needs further investigation. No significant associated markers with heading date were found in the immediacy of *Hd3a* (2939760–2,942,696) or *RFT1* (2926823–2,928,474) located in chromosome 6.

Some novel loci associated to heading date described in this study are promising according to the difference in heading date of cultivars carrying one or the other allele. In these sense, DH-7 and DH-8, both in the same loci in chromosome 10, appeared to be good candidates as the difference in heading date of cultivars carrying one or the other allele is 7 and 5 days respectively (Additional file 4: Table S2). DH-7 and DH-8 accounted for the 7.2 and 6.7% respectively of the explained variance (Table 3). Whether these loci are associated to photoperiod regulation of flowering remains unknown and further investigation is needed.

Several loci were identified associated with panicle length. We didn’t find any functionally characterized gene in the vicinity of 7 of these loci and we could considerer that they represent novel associated sites with panicle length. Some of the detected SNPs were close to genes that participate in panicle elongation but that also affect other parts of the plant. This is the case of PL-8 that mapped at a distance of 0.5 Mb of *SALT-RESPONSIVE ERF1* (*SERF1*), is a transcriptional factor that acts as negative regulator of grain filling. Mutants carrying a null *SERF1* allele resulted in larger grains and altered panicle length [26]. *OsEBS*, at 0.9 Mb of distance from PL-10, produces higher spikelet number in the panicles, leading to an increase in total grain yield although also affects plant height and leaf size [4]. Introgression lines carrying the *Oryza rufipogon* allele of *OsEBS* also produce longer panicles than wild type [4]. *Oryza sativa dwarf rice with overexpression of gibberellin-induced gene (OsDOG),* involved in gibberellin homeostasis, was located at 0.8 Mb from PL-12 and less than 1.2 Mb from H-2. OsDOG is an A20/AN1 zinc-finger protein that negatively regulates gibberellin-mediated cell elongation in rice. Transgenic plants expressing *OsDOG* exhibited dwarf phenotypes and shorter panicles that did not completely emerge from the leaf sheath due to deficient cell elongation [18]. *OsDOG* was also found associated with panicle length in a recent study of *japonica* rice grown under permanent flooding conditions [32]. *LAGGING GROWTH AND DEVELOPMENT 1* (*LGD1*) is also close to PL-13. The *lgd1* mutant displays pleotropic effects in rice, showing slow growth, reduced tiller number and plant height, altered panicle architecture and reduced grain yield [30]. On the other side, PL-6 localized close to *rice panicle 3* (*rip-3*), coding for a putative α-tubulin protein, which expression was observed in all the reproductive organs of rice panicle, but not in other parts of the plant. Interestingly, *rip-3* acts, supposedly, as suppressor of panicle elongation in the regions of high growth and in periods of water deficit [27]. Panicle length is often associated with yield as long panicles may produce higher number of spikelets and, thus, higher number of grains [17]. But in our collection, no correlation could be found between panicle length and the number of grains per panicle (Table 2). This is in agreement with the fact that no significant markers were detected in common when analysing both panicle length and number of grain per panicle.

## Conclusion

In this association study, we have identified molecular markers related to important agronomic traits among cultivars adapted to temperate photoperiod conditions and, thus, they have a direct application in breeding programs. Some of these markers co-localizing with known genes or QTLs, such as *DEP2*, *LRK1* or *LGD1*, validate our methodology while the study provided novel molecular markers that can be of help to elucidate the complicated genetic mechanism controlling important agronomic traits in *japonica* rice, as flowering regulation under log day conditions.

## Methods

### Plant material and growing conditions and Phenotyping

A *japonica* type rice collection composed by 193 cultivars was obtained from different sources [24]: The International Rice Research Institute (IRRI, Philippines), U.S. National Plant Germplasm System (NPGS, USA), Rice Genome Resource Center (RGRC, Japan), Copsemar and Instituto Valenciano de Investigaciones Agrarias (IVIA, Spain). Seeds of different cultivars were cultivated in two locations during 2015 and 2016 summer seasons, Tancat de Malta and Finca de Raga, in Valencia rice growing area in Spain. Plantlets were germinated in pots and manually transplanted to soil in double rows of 20 plants in May and harvested in September. Fields were continuously irrigated by flooding and drained two weeks before harvesting. Two additional batches of plants were grown separately during 2017 summer season in a greenhouse under natural day condition.

Height and heading time were measured in the field. Heading time was measured as 50% of panicle emergence in each variety. Three plants per cultivar were collected from each location and panicle number and panicle length were scored on three representative panicles for each plant. The number of grains per panicle was measured in three different panicles per plant from plants grown in the greenhouse.

### Statistical analysis

Phenotypic data analyses (means, coefficients of variation and histograms of frequency distribution) were performed using Statgraphics Plus. Correlations between trait values were examined by Pearson’s correlation coefficient test using statistical software R (https://www.r-project.org/). Broad sense heritability was calculated using linear regression between mean value for variety at 2015 and 2016 at Malta field and mean value for variety at 2015 and 2016 at Copsemar, using lm command of R. For grain number regression was performed between plants at different sides of the green house.

### Marker trait association analysis

For the marker trait association analysis, a 1713 SNP panel obtained in a previous study was used [24]. This panel was generated from a custom Infinium SNP genotyping array compiling 2697 SNP representative of *japonica* rice cultivated under long day conditions after removing SNP not present in at least 75% of varieties of the rice core pool, or showing a MAF lower than 5%. Association between phenotype and markers was analyzed using Tassel software (version 5.2.34) [2]. Mixed linear model (MLM) was used with kinship control (K) and with structure control (Q) to avoid spurious associations [41].

K and Q matrix were obtained from a 948 SNP panel, this one was created after filtering the 1713 SNP panel according to the previously estimated linkage disequilibrium decay distance extension for *japonica* subset from the collection [24]. Kinship matrix was computed with Tassel and two Q matrices were created. First one was obtained using STRUCTURE 2.3.4 software [22] retaining the membership value of three of four groups, being four the optimum estimated number of groups for this collection [24]. The latter was generated after performing a Principal Component Analysis with Tassel and by screen plot where 7 principal components were retained, which accounted for 49% of total variance (Additional file 5: Table S4). The threshold value for significant association marker-phenotype was set at *p* < 10^− 3^ due to the effectivity of the use of MLM with correction to avoid spurious associations. Fdr values were calculated using “qvalue” package 1.99.1. Manhattan plots were drawn using modified “qqman” package (version 0.1.4) for R.

To search for functionally characterized genes harboring the detected SNPs or within a 1.2 Mb interval, we looked at the Nipponbare reference annotation database (Os-Nipponbare-Reference-IRGSP-1.0) and explored the OGRO database [35] and, in addition, the Q-TARO database [39] in a search for QTLs in the same trait category.

## Additional files

Additional file 1: **Table S1**. Mean values of height, days to heading, panicle length, number of panicles and grains per panicle from the different assays in both locations. SD, standard deviation. (XLSX 64 kb)

Additional file 2: **Figure S1**. Boxplot showing the distribution of data scored for days to heading, height, number of panicles and panicle length in both locations, Copsemar and Malta, during 2015 and 2016. (PPTX 75 kb)

Additional file 3: **Table S3**. Number of alleles carried by the 20 plants with the highest and the lowest value for each trait. Rr: heterozygous; NN: indetermination. (XLSX 13 kb)

Additional file 4: **Table S2**. Mean phenotypic values of cultivars carrying different alleles in the significant associated markers. (XLSX 16 kb)

Additional file 5: **Table S4**. PCA. Proportion of the total and cumulative proportion of each principal component. (XLSX 16 kb)

## Abbreviations

DH: Heading date
GN: Number of grains per panicle
GWAS: Genome wide association study
H: Plant height
LD: linkage disequilibrium
MLM: Mixed linear model
PCA: Principal component analysis
PL: Panicle length
PN: Number of panicles
