# Supplementary figures and images for: Genome-wide association study of agronomic traits in rice cultivated in temperate regions
Source: BMC Genomics. 2018 Sep 25;19:706. doi: 10.1186/s12864-018-5086-y (PMC6156875; doi:10.1186/s12864-018-5086-y)

## Slide 1
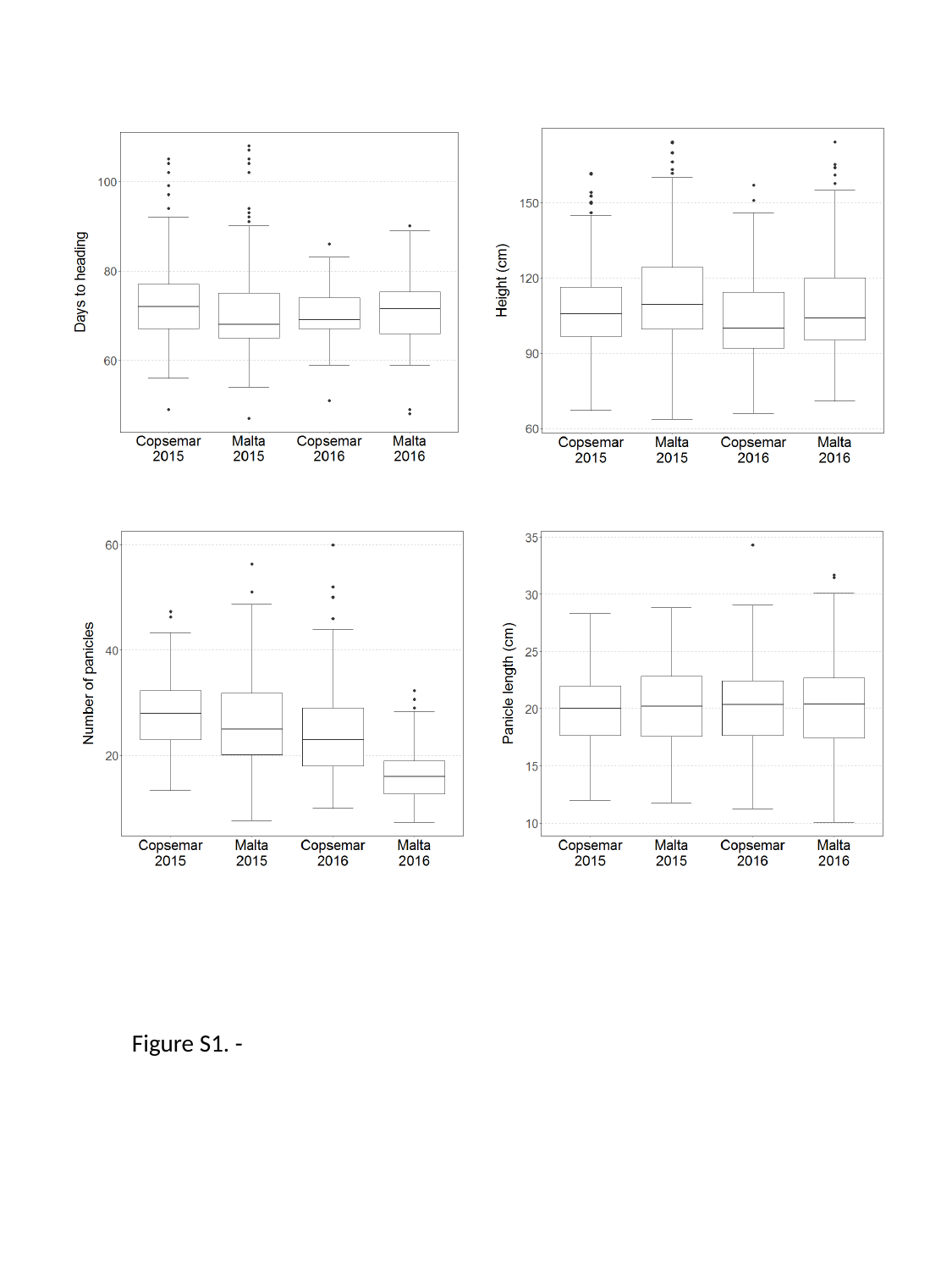

Figure S1. -

Supplement: Supplementary file 2 — Figure S1. Boxplot showing the distribution of data scored for days to heading, height, number of panicles and panicle length in both locations, Copsemar and Malta, during 2015 and 2016. (PPTX 75 kb) [file 12864_2018_5086_MOESM2_ESM.pptx]
